# Knee Arthroplasty: Disabilities in Comparison to the General Population and to Hip Arthroplasty Using a French National Longitudinal Survey

**DOI:** 10.1371/journal.pone.0002561

**Published:** 2008-07-02

**Authors:** Agnes Dechartres, Isabelle Boutron, Remy Nizard, Serge Poiraudeau, Carine Roy, Gabriel Baron, Philippe Ravaud, Jean-François Ravaud

**Affiliations:** 1 INSERM, U738, Paris, France; 2 Université Paris 7, UFR de Médecine, Paris, France; 3 AP-HP, Hôpital Bichat, Département d'Epidémiologie, Biostatistique et Recherche Clinique, Paris, France; 4 Assistance Publique-Hôpitaux de Paris (AP-HP), Hôpital Lariboisière, Service d'Orthopédie, Paris, France; 5 Université Paris VII, Paris, France; 6 Assistance Publique-Hôpitaux de Paris (AP-HP), Hôpital Cochin, Service de Médecine Physique et de Réadaptation, Paris, France; 7 Université Paris V, Paris, France; 8 INSERM, U750-CERMES, Paris, France; 9 Institut Fédératif de Recherche sur le Handicap, Paris, France; University Medical Center Rotterdam, Netherlands

## Abstract

**Background:**

Knee arthroplasty is increasing exponentially due to the aging of the population and to the broadening of indications. We aimed to compare physical disability and its evolution over two years in people with knee arthroplasty to that in the general population. A secondary objective was to compare the level of disabilities of people with knee to people with hip arthroplasty.

**Methodology/Principal Findings:**

16,945 people representative of the French population were selected in 1999 from the French census and interviewed about their level of disability. This sample included 815 people with lower limb arthroplasty. In 2001, 608 of them were re-interviewed, among whom 134 had knee arthroplasty. Among the other participants re-interviewed, we identified 68 who had undergone knee arthroplasty and 145 hip arthroplasty within the last two years (recent arthroplasty). People with knee arthroplasty reported significantly greater difficulties than the general population with bending forward (odds ratio [OR] = 4.7; 95% confidence interval [CI]: 1.7, 12.6), walking more than 500 meters (OR = 6.0; 95% CI: 1.5, 24.7) and carrying 5 kg kilograms for 10 meters (OR = 4.6; 95% CI: 1.3, 16.4). However, the two years evolution in disability was similar to that in the general population for most activities. The level of mobility was similar between people with recent knee arthroplasty and those with recent hip arthroplasty. Nevertheless, people with recent knee arthroplasty reported a lower level of disability than the other group for washing and bending forward (OR = 0.3; 95% CI: 0.1, 0.6 and OR = 0.4; 95% CI: 0.1, 0.9, respectively).

**Conclusions/Significance:**

People with knee arthroplasty reported a higher risk of disability than the general population for common activities of daily living but a similar evolution. There was no relevant difference between recent knee and hip arthroplasties for mobility.

## Introduction

The prevalence of knee replacement is increasing exponentially because of the aging of the population but also due to the broadening of indications [1]. The projections between 1996 and 2030, based on changes expected in the population's age profile, foresee an increase of about 85% in total knee replacements in United States [2]. Data on the level of disability and evolution over time of this increasing population are needed for health service planning and budgeting resources and for better informing patients about their potential difficulties after the surgery. Cohort studies have confirmed that mobility and relief of pain are improved after knee arthroplasty [3]–[7], but no study has compared the remaining level of disability after the replacement to that in the general population. Results of a French longitudinal community-based study, the “Handicap, Incapacité, Dépendance” (Handicap, Disability and Dependence) or HID survey, showed that people with hip arthroplasty had a higher level of disability than the general population [8]. Most published results suggest that improvement after knee arthroplasty is lower than after hip arthroplasty [3]–[5], [9]–[14]. Actually, many prospective studies [3]–[5], [9]–[14] reported that postoperative improvement in pain relief and physical function were greater for subjects with hip arthroplasty than for those with knee arthroplasty. Fitzgerald et al. [15] compared data on physical activities of people with hip and knee arthroplasty to normative data and showed physical function to be remarkably similar at 6 and 12 months postoperatively between patients undergoing either hip or knee arthroplasty. However, to our knowledge no published data has provided a comparison of activity limitations and evolution of disability between people with knee arthroplasty and those with hip arthroplasty in a national representative sample.

## Methods

### Objectives

The primary objective of our study was to compare physical disabilities and their evolution over two years between people with knee arthroplasty and the general population with use of the two-year follow-up data of the HID survey. A secondary objective was to compare activity limitations and their evolution over two years between subjects with recent (less than two years) knee arthroplasty and those with similarly recent hip arthroplasty.

### Participants

The data constituting the basis of this report were collected from the HID survey, a national longitudinal community-based survey undertaken by the French National Institute of Statistics and Economic Studies (INSEE) to describe disability and handicap in France. The target population included residents in all French households (n = 57.4 million) including children. This survey methodology is described in detail elsewhere [8], [16], [17].

Briefly, a two-stage method was used according to United Nations recommendations [18] to set up the cohort in 1999. For the first stage, a representative sample of census districts (approximately 600 inhabitants per district) was selected. During the census taking, enumerators gave these households the standard forms of the 1999 French population census and an additional questionnaire concerning daily life and health. This screening questionnaire allowed for classifying people into 6 groups of increasing probability of presumed disability. This first 1999 phase concerned approximately 417,500 people and had an 86% response rate.

**Figure 1:**
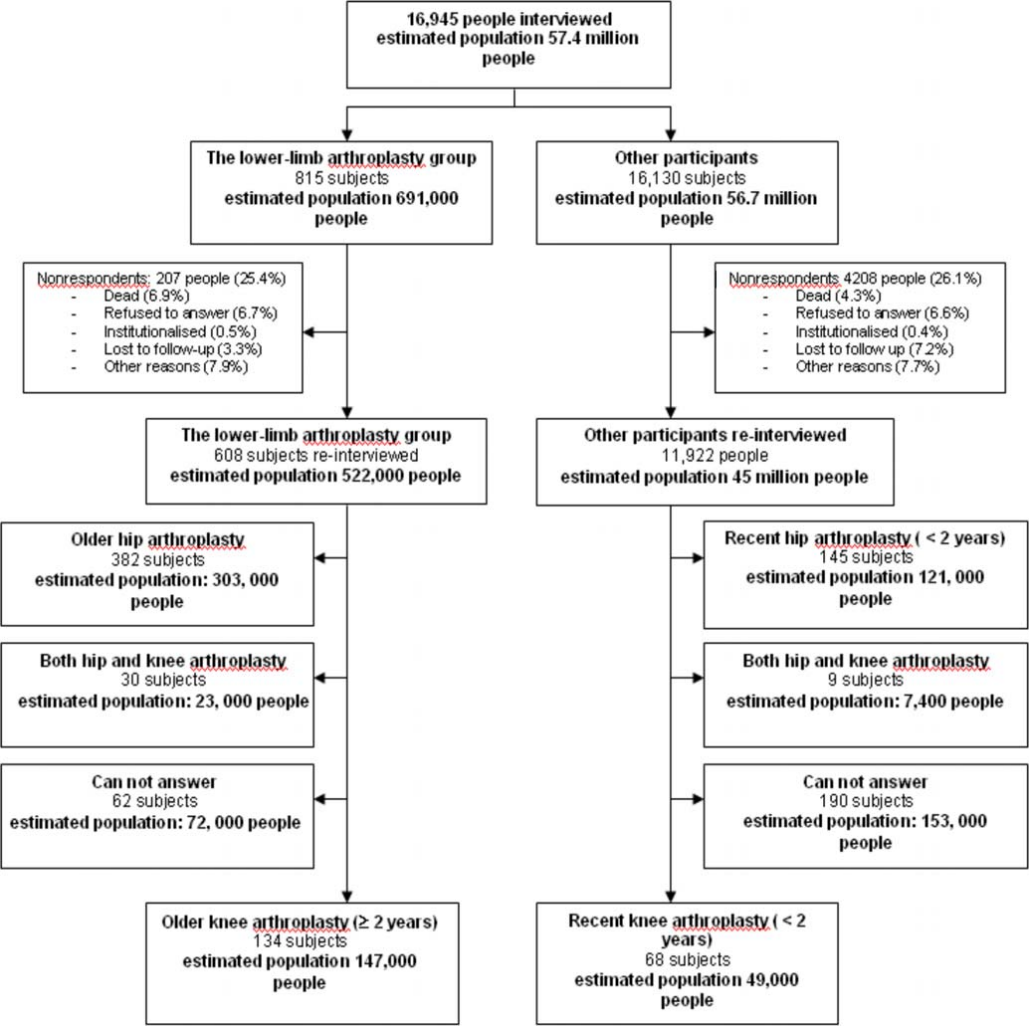
Study population of the HID survey.

For the second stage, we selected the population by randomization, using disproportional sampling, with a high sampling rate for the most severely disabled group and a minimum sampling rate for people without daily living restrictions (the largest group). Each of the resulting groups was allocated a specific sampling coefficient that increased with the probability or severity of the presumed handicap. The sample design allowed for weighting the data to estimate representative results at a national level[8]. This cohort included 16,945 subjects representative of the French population living at home, 815 of whom reported having undergone lower-limb arthroplasty (i.e., estimated 691,000 people in the French noninstitutionalised population consistent with the result of another French study[19]). For the two year follow-up survey, 12,530 people were re-interviewed, among whom were 608 of the 815 subjects with lower-limb arthroplasty and 11,922 of the 16,130 other participants. Reasons for no interview are shown in figure 1. Characteristics of nonrespondents are described elsewhere and show that they were significantly older and more disabled for walking and bending forward than respondents [8].

The questionnaire included several items to identify people with hip and knee arthroplasty. There were both open-ended questions concerning health problems and 2 specific questions concerning lower limb arthroplasty “Do you have hip arthroplasty” and “Do you have knee arthroplasty”. Among the 608 subjects with lower-limb arthroplasty identified in 1999, 134 (i.e., estimated 147,000 people in the French noninstitutionalised population) had received knee arthroplasty (older knee arthroplasty), 382 hip arthroplasty, 30 both hip and knee arthroplasty and 62 had uncertain data. Among the general population re-interviewed, 68 and 145 (i.e., estimated 49,000 and 121,000 people, respectively, in the French noninstitutionalised population) declared having undergone knee and hip arthroplasty since 1999 (recent knee and hip arthroplasty). To validate the quality of our data and particularly to avoid the possibility that recent arthroplasty was misclassified as older arthroplasty, one member of our team systematically assessed the chronic diseases reported during the 1999 survey and the 2001 survey by the people identified as having recent arthroplasty. Consequently, 202 people with knee arthroplasty (i.e., estimated 196,000 people in the French noninstitutionalised population) were selected.

### Data collection

Two computer-assisted personal interviews were conducted according to a standardized protocol from November 1999 to January 2000 by 442 trained interviewers for the 1999 survey and from October 2001 to January 2002 by 408 trained interviewers for the 2001 survey. During each interview, respondents were asked about their impairment, disability, social participation and help. For the current study, we used data related to participants' disability. Respondents were asked to indicate the degree of difficulty or the need for help in five defined areas: activities involving personal care, mobility, housekeeping, and cognitive and sensorial abilities. Answers were presented as a 5-point Likert scale, ordered by growing probability of disability, from “no disability” to “impossible without help”. Respondents also indicated their walking distance: no more than 100 meters, 300 meters, 500 meters and 1000 meters. For the 2001 survey, conditions were considered improved for each task if the answer decreased by at least one category and worse if the answer increased by at least one category. If the answers did not differ between 1999 and 2001, we considered that there was no change in disability for the studied task. The complete questionnaire will be available at the following address: http://ifr-handicap.inserm.fr/HID/ACCUEIL_HID_NEW.HTM

### Ethics

This study was planned as a research project. This study was performed in an intense collaboration with the French National Institute of Statistics. This study was declared of public interest by the CNIS (“Conseil National d'Information Statistique”) and was approved by the CNIL (“Commission Nationale de l'Informatique et des Libertés”, French law number 78-17). According to the French law, written inform consent was not required for this type of study.

### Statistical methods

The study design allowed for weighting the data to estimate representative results at a national level. To calculate statistical parameters and their 95% confidence intervals [CI], we used SAS procedures specific for handling complex sample designs to obtain correct variance estimates (SAS version 9.1, PROC SURVEYFREQ, PROC SURVEYMEAN, PROC SURVEYREG and PROC SURVEYLOGISTIC [20].

To take into account attrition bias, we built a propensity score to assess the probability of nonresponse. To do so, we performed a logistic regression with the 2001 responses to the survey used as the dependent variable. The covariates were the 1999 demographic, economic, and educational variables as well as the level of disability in performing common activities of daily living from the 1999 survey. The logistic regression analysis was used to determine the probability of nonresponse (from 0 to 1), the propensity score, for each patient in the data set. Then, multiple logistic regression models adjusted for age, sex, education, number of chronic conditions and probability of 2001 nonresponse allowed for estimating odds ratios [ORs] and their 95% confidence interval [CI] for 1) the level of disability in all subjects with knee arthroplasty compared to the general population in 2001; 2) the evolution of disability (i.e., worsening) in subjects with knee arthroplasty identified in 1999 compared to the general population; and 3) the comparison of disability and its evolution over two years (i.e., worsening) between subjects with recent knee arthroplasty and those with recent hip arthroplasty. To compare the level of disability, the dependent variable was ‘reporting at least some difficulties for each activity of daily living’. To compare the evolution of disability, we decided to focus on the worsening of disability as defined above. This choice was based on the descriptive analysis of recent hip and knee arthroplasty, which showed a higher rate of worsening disability than improved condition. Because the delay since the procedure is not known, evolution of disability in our analysis does not reflect evolution since surgery. Thus, analysis of evolution refers to worsening disability for all analysis. Separate models were created for each activity of daily living. All data analyses involved use of the SAS statistical software (version 9.1, SAS Institute).

## Results

### Demographic characteristics

The knee arthroplasty population comprised 202 subjects, for an estimated 196,000 people in the French noninstitutionalised population (95% CI: 124,000, 269,000). The overall prevalence of knee arthroplasty in France is, consequently, estimated at 0.43% (95% CI 0.27, 0.59). The annual incidence of knee arthroplasty in France is estimated at 0.05% (95% CI: 0.04%, 0.06%).

The mean age of subjects with knee arthroplasty was 70.2 years (95% CI: 64.8, 75.6; minimum 20, maximum 96); 40.8% of subjects were 75 years or older (95% CI: 24.2, 57.4). Most people were retired: 66.5% (95% CI: 38.3, 94.7). Women represented 52.7% of the study population (95% CI: 32.1, 73.3). Primary school was the highest level of education for 83.2% of people with knee arthroplasty (95% CI: 72.7, 93.7).

### Disability and self-reported health status for all people with knee arthroplasty

Subjects with knee arthroplasty claimed 3.6 chronic conditions, on average (95% CI: 3.3, 3.9; minimum 0, maximum 11). Activity limitations concerned mainly the areas of mobility: 52.2% (95% CI: 32.1, 72.3) could not walk more than 500 meters; 73.6% (95% CI: 61.9, 85.4) reported at least some difficulties in bending forward and picking up something; 53.3% (95% CI: 31.5, 75.0) had at least some difficulties climbing up and down stairs; and 70.1% (95% CI: 57.0, 83.2) at least some difficulties carrying 5 kilograms for 10 meters. Self-reported health was poor or very poor for 37.2% of subjects (95% CI: 6.6, 74.9).

After adjustment for age, sex, education, chronic conditions and probability of nonresponse, subjects with knee arthroplasty reported significantly greater difficulties than the general population for bending forward and picking up something (OR = 4.7; 95% CI: 1.7, 12.6), walking more than 500 meters (OR = 6.0; 95% CI: 1.5, 24.7), carrying 5 kilograms for 10 meters (OR = 4.6; 95% CI: 1.3, 16.4) and cutting toenails (OR = 4.1; 95% CI: 1.2, 13.8) (table 1). They also more often reported the use of technical devices to walk (OR = 3.6; 95% CI: 1.7, 7.2). There was a tendency for self-reported health probability to be worse than in the general population (OR = 2.0; 95% CI: 0.9, 4.7).

**Table 1:** Disability according to domains of disability, for all subjects with knee arthroplasty (202 subjects) compared to the other participants (11,510 subjects) in 2001, after adjustment for age, sex, education, number of chronic conditions and probability of nonresponse. Adjusted Odds ratio [Adj-OR] and 95% confidence interval [CI].

Domains of disability	Subcategory	Adj-OR*	95% CI
Self-care	Washing	0.7	[0.3–1.4]
	Dressing	0.6	[0.3–1.4]
	Cutting toenails	**4.1**	**[1.2–13.8]**
	Using the toilet	0.9	[0.4–2.0]
Mobility	Getting in and out of a bed	1.1	[0.6–2.2]
	Getting in and out of a chair	**2.1**	**[1.05–4.4]**
	Climbing up and down stairs	1.7	[0.7–4.8]
	Bending forward and picking up something	**4.7**	**[1.7–12.6]**
Walking-distance limitation	≤100 meters	1.5	[0.7–3.3]
	≤300 meters	1.3	[0.6–2.6]
	≤500 meters	**6.0**	**[1.5–24.7]**
	≤1000 meters	**5.9**	**[1.6–21.9]**
Shopping		3.3	[0.9–12.9]
Carrying 5 kilograms for 10 meters		**4.6**	**[1.3–16.4]**
Heath status	Having fair, poor or very poor self-reported health	2.0	[0.9–4.7]
Use of technical devices to walk		**3.6**	**[1.7–7.2]**

* Adj-OR, adjusted OR.

Significant ORs are in bold.

The dependent variable is «reporting at least some difficulties for each activity of daily living».

### Evolution of disability declared by people with knee arthroplasty for more than 2 years

Worsening disability was mainly declared for mobility: of subjects with knee arthroplasty identified in 1999, 49.3% (95% CI: 19.9, 78.7) declared worsening disability for bending forward and picking up something, 47.1% (95% CI: 19.4, 74.8) for cutting toenails, 21.4% (95% CI: 7.4, 35.4) for shopping, 17.9% (95% CI: 5.5, 30.3) for climbing up and down stairs and 17.7% (95% CI: 5.6, 29.9) for walking-distance limitation.

After adjustment for age, sex, education, chronic conditions and probability of nonresponse, the evolution was similar between subjects with knee arthroplasty for more than 2 years and the general population, except for bending forward and picking up something (OR = 6.4, 95% CI: 1.1, 38.3), cutting toenails (OR = 6.4, 95% CI: 1.1, 37.6) and using the toilet (OR = 0.2, 95% CI: 0.05, 0.9) (Table 2).

**Table 2:** Disability worsening over two years according to domains of disability, for subjects with knee arthroplasty identified in 1999 (134 subjects) compared to the other participants (11,510 subjects), after adjustment for age, sex, education, chronic conditions and probability of nonresponse.

Domains of disability	Subcategory	Adj-OR	95% CI
Self-care	Washing	0.6	[0.2–1.6]
	Dressing	0.7	[0.3–1.9]
	Cutting toenails	**6.4**	**[1.1–37.6]**
	Using the toilet	**0.2**	**[0.05–0.9]**
Mobility	Getting in and out of a bed	0.6	[0.2–1.7]
	Getting in and out of a chair	1.7	[0.7–4.2]
	Climbing up and down stairs	0.9	[0.4–2.1]
	Bending forward and picking up something	**6.4**	**[1.1–38.3]**
Walking-distance limitation*		1.3	[0.5–3.1]
Shopping		1.2	[0.6–2.4]
Carrying 5 kilograms for 10 meters		0.8	[0.4–1.8]
Heath status		0.4	[0.2–1.2]
Use of technical devices to walk		1.5	[0.5–4.3]

Adjusted Odds ratio [Adj-OR] and 95% confidence interval [CI].

Significant ORs are in bold.

* Worsening walking-distance limitation was defined by the decrease of a class (i.e., <100, 100–300, 300–500, 500–1000) to another.

### Comparison between subjects with recent knee arthroplasty and those with recent hip arthroplasty

**Figure 2:**
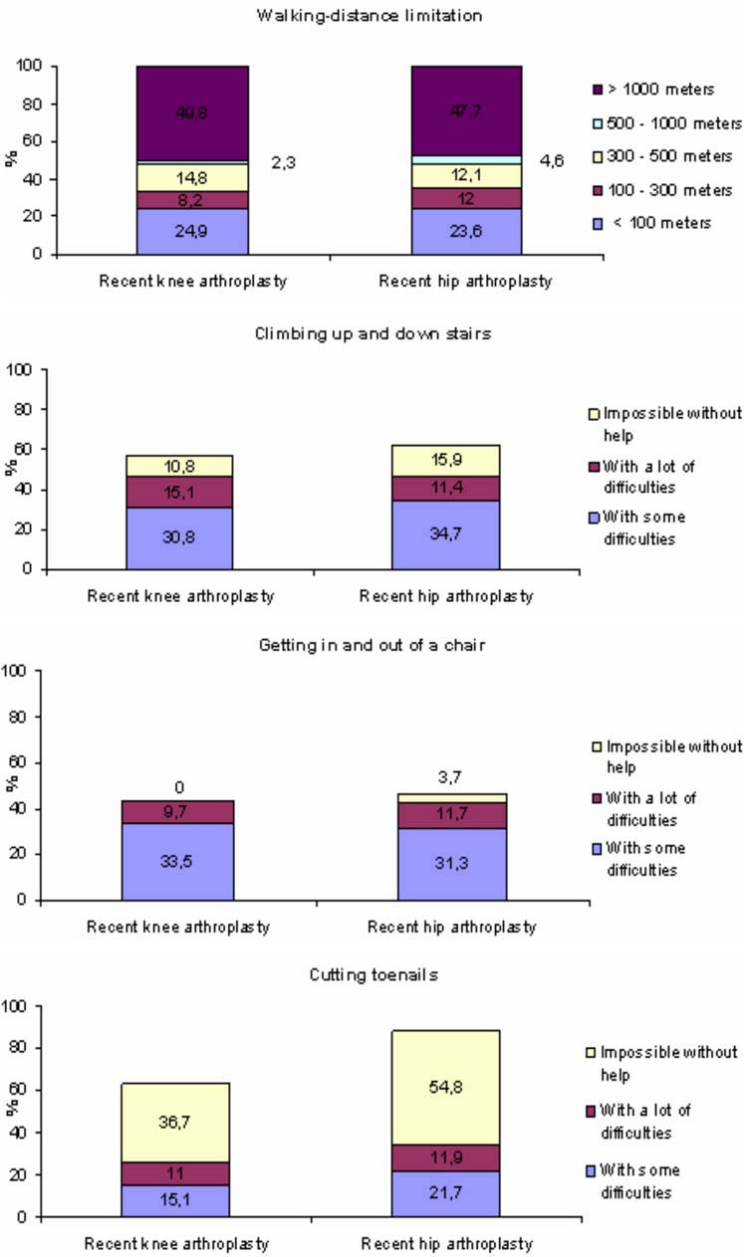
Disability level for people with recent knee arthroplasty and those with recent hip arthroplasty for some activities of daily living in 2001.

For this comparison, we focused only on recent arthroplasty. Subjects with recent knee arthroplasty tended to be younger than people with recent hip arthroplasty (69.8 years *vs.* 73.1 years, p = 0.07). The number of chronic conditions and proportion of women were not significantly different between the 2 groups. Figure 2 provides a description of the level of difficulties for the 2 groups for four common activities of daily living. For walking-distance limitation and climbing stairs, overall, people with recent knee or hip arthroplasty reported the same level of difficulties. However, people with knee arthroplasty reported less difficulties for washing and bending forward than those with hip arthroplasty.

Evolution over two years was largely characterized by no change for both people with recent knee or hip arthroplasty (table 3).

**Table 3:** Evolution of disability for people with recent (less than 2 years) knee arthroplasty (68 subjects) and for those with recent hip arthroplasty (145 subjects).

Domain	Subcategory	Worsening: % [95% CI]		No Change: % [95% CI]		Improvement: % [95% CI]	
		Knee	Hip	Knee	Hip	Knee	Hip
Self-care	Washing	18.8[7.9–29.7]	35.2[25.2–45.2]	73.8[61.4–86.2]	52.0[41.7–62.3]	7.4[0–14.8]	12.8[6.3–19.3]
	Dressing	18.1[7.7–28.5]	34.6[24.7–44.6]	69.5[56.7–82.3]	47.5[37.2–57.8]	12.4[3.2–21.6]	17.9[10.5–25]
	Cutting toenails	30.5[18.0–43.0]	31.3[22.1–40.5]	53.0[38.9–67.2]	55.2[45.0–65.3]	16.4[6.1–26.8]	13.5[6.6–20.4]
	Using the toilet	8.8[0.9–16.8]	16.4[9.4–23.4]	87.8[78.6–96.9]	77.0[69.2–84.8]	3.4[0–8.4]	6.6[2.7–10.5]
Mobility	Getting in/out of a bed	20.6[10.1–31.1]	29.6[19.8–39.3]	58.8[45.2–72.5]	59.2[49.2–69.3]	20.6[9.6–31.5]	11.2[6.3–16.2]
	Getting in/out of a chair	26.1[13.7–38.5]	27.8[18.7–36.9]	54.4[40.2–68.5]	58.6[48.7–68.5]	19.5[8.8–30.2]	13.6[7.8–19.4]
	Bending forward	36.8[22.3–51.3]	32.3[22.6–41.9]	53.0[38.1–67.8]	48.8[37.8–59.7]	10.2[2.2–18.3]	18.9[11–26.7]
	Climbing stairs	23.7[11.4–36.1]	28.3[19.4–37.2]	45.2[30.0–60.3]	49.8[39.1–60.5]	31.1[17–45.2]	21.9[13–30.7]
Walking		39.6[25.2–53.9]	29.6[18.9–40.2]	42.0[27.5–56.5]	51.6[40.1–63.1]	18.4[8.2–28.7]	18.8[8.9–28.6]
Shopping		28.0[15.3–40.6]	21.9[13.7–30.0]	53.4[39.0–67.9]	64.7[54.8–74.6]	18.6[8.1–29.0]	13.5[7.1–19.8]
Carrying 5 kg for 10 m		33.3[19.9–46.7]	33.4[23.6–43.1]	49.8[35.5–64.1]	51.1[40.7–61.4]	16.9[7.0–26.8]	15.6[8.1–23.0]
Health status		21.0[9.3–32.8]	20.3[12.6–28.1]	45.9[31.0–60.8]	57.5[47.3–67.7]	33.0[18–47.9]	22.2[14–30.2]
Use devices to walk		15.9[5.3–26.4]	21.6[12.2–31.0]	73.6[61.2–86.0]	72.7[62.9–82.5]	10.5[2.4–18.6]	5.8[1.2–10.3]

Percentages and 95% confidence interval [CI].

After adjustment for age, sex, education, chronic conditions and probability of nonresponse, subjects with recent knee arthroplasty reported significantly less difficulties than those with recent hip arthroplasty for washing (OR = 0.3; 95% CI: 0.1, 0.6), dressing (OR = 0.3; 95% CI: 0.1, 0.5), bending forward (OR = 0.4; 95% CI: 0.1, 0.9) and cutting toenails (OR = 0.2; 95% CI: 0.1, 0.5) (table 4). After adjustment, worsening disability was less often reported by subjects with recent knee arthroplasty for washing (OR = 0.4; 95% CI: 0.1, 0.9) and dressing (OR = 0.3 95% CI: 0.1, 0.9) than by subjects with recent hip arthroplasty (table 4).

**Table 4:** Disability and evolution (i.e., worsening) over two years according to domains of disability, for subjects with recent knee arthroplasty (68 subjects) compared to those with recent hip arthroplasty (145 subjects) adjusted for age, sex, education, chronic conditions and probability of nonresponse.

		Disability$		Worsening	
Domains of disability	Subcategory	Adj-OR	95% CI	Adj-OR	95% CI
Self-care	Washing	**0.3**	**[0.1–0.6]**	**0.4**	**[0.1–0.9]**
	Dressing	**0.3**	**[0.1–0.5]**	**0.3**	**[0.1–0.9]**
	Cutting toenails	**0.2**	**[0.1–0.5]**	0.7	[0.3–1.7]
	Using the toilet	0.4	[0.1–1.5]	0.4	[0.1–1.7]
Mobility	Getting in and out of a bed	0.6	[0.2–1.4]	0.5	[0.2–1.5]
	Getting in and out of a chair	0.9	[0.4–2.1]	1.3	[0.5–3.3]
	Climbing up and down stairs	0.9	[0.3–2.3]	0.7	[0.3–1.9]
	Bending forward and picking up something	**0.4**	**[0.1–0.9]**	1.0	[0.4–2.4]
Walking-distance limitation	≤100 meters	1.1	[0.4–3.0]		
	≤300 meters	0.9	[0.3–2.2]		
	≤500 meters	1.4	[0.5–3.6]		
	≤1000 meters	1.1	[0.4–2.9]		
	Worsening*			1.3	[0.5–3.3]
Shopping		0.5	[0.2–1.3]	0.9	[0.4–2.2]
Carrying 5 kg for 10 m		0.6	[0.2–1.8]	0.9	[0.4–2.1]
Heath status	Having fair, poor or very poor self-reported health	0.9	[0.3–2.6]	1.5	[0.5–4.4]
Devices to walk	Use of technical devices to walk	0.5	[0.2–1.2]	0.7	[0.2–2.1]

Adjusted Odds ratio [Adj-OR] and 95% confidence interval [CI].

$ The dependent variable is «reporting at least some difficulties» for each activity of daily living.

* The term worsening was used to assess overall worsening of walking-distance limitation.

Significant ORs are in bold.

## Discussion

This study compared the disabilities of people with knee arthroplasty and their evolution over two years to the general population and to people with hip arthroplasty, on a national representative sample. Our results showed that, despite a higher level of disability for several activities of daily living reported by people with knee arthroplasty, the evolution in disability over two years was similar to that in the general population for most tasks. Moreover, people with recent knee arthroplasty did not report more disabilities or worsening of these disabilities than people with recent hip arthroplasty for activities involving mobility; they reported an even lower level of disability and less worsening of disability for self-care activities.

Many studies have reported significant improvements in ability after knee arthroplasty [3]–[5], [7], [21]–[24]. Only a few have described activity limitations for people with knee arthroplasty [9], [23], [25], [26]: Hawker *et al.* [23] showed, in a community-based survey that 70.2% of subjects with knee arthroplasty had difficulties going upstairs, 66.3% bending to the floor, and 56.9% shopping, and only 38.8% could walk more than 10 blocks; Weiss *et al.* [25] reported that 72% could not kneel and 40% had difficulties carrying heavy objects; and Rissanen *et al.* [9] reported that only 34.7% and 16.7% had no problem with walking and negotiating stairs, respectively, at 24 months. Our results support these studies showing that people with knee arthroplasty do not reach the level of mobility reported by the general population. Evolution of these disabilities over 2 years was not different from the general population except for bending forward and cutting toenails that had a higher risk of worsening. People with knee arthroplasty have less risk of worsening disability for using the toilet than the general population. This result is surprising and unexplained; it could only be related to multiple testing. Our results suggest that mean age at surgery seems to increase since people with recent and older knee arthroplasty have nearly the same mean age during the follow-up visit. This result may be related to the selection of older people for knee replacement.

Results of surveys comparing hip and knee arthroplasty are inconsistent. Many studies report larger pain relief and improvement in physical function for people with hip arthroplasty than for people with knee arthroplasty [3]–[5], [9]–[14]; others did not find differences between the 2 groups [15], [22]. These studies were mainly performed in a few specialized centers by highly qualified surgeons, and their results should not be generalizable to the entire arthroplasty population [27]–[29]. Our survey, involving a national representative sample, found that subjects with recent knee or hip arthroplasty did not differ in level of disability in mobility activities and evolution over two years. This finding confirms the results of Fitzgerald *et al.* [15] and Ritter *et al.* [22], who showed no difference in physical functioning between people with hip arthroplasty and those with knee arthroplasty 12 months postoperatively and two years later, respectively. A possible explanation concerning the lack of difference in mobility between people with knee arthroplasty and those with hip arthroplasty may be that knee replacements are performed for a lower level of disability than are hip replacements, which implies better outcomes for the former patients [21], [30]. The higher risk of disability reported by people with hip arthroplasty for self-care activities and bending forward than for people with knee arthroplasty could be related to the recommendations given to patients after hip arthroplasty to limit activities to reduce the risk of dislocation. These recommendations concern mainly movements to avoid when washing, dressing and bending forward.

### Limitations

Our study has some limitations. First, the design of this study is based on self-reported conditions. Self reporting is important to assess disabilities [31], [32] but could imply possible under-reporting of arthroplasty by the participants interviewed. We also missed other important information about the different indications for surgery, the timing of procedures, the surgical procedures (total or partial knee replacement), the rehabilitation programmes, the postoperative complications and the revision rates. Second, nonrespondents in the general population could have undergone an arthroplasty within the two years, which would also contribute to possible underestimation of arthroplasty prevalence. Third, our study concerned only French noninstitutionalised people, which would lead to both underestimation of arthroplasty prevalence and disability, because, typically, disabled people tend to live in institutions. Moreover, we can hypothesize that the more disabled and health-impaired people are not undergoing arthroplasty. Although France has no register of knee arthroplasty, our results of prevalence are consistent with those of another survey performed in France in 1999 [19] that identified an estimated 270,000 people with knee arthroplasty. The comparison between people with recent hip arthroplasty and those with recent knee arthroplasty should be interpreted carefully since these results may differ for a longer follow-up. As already studied, the delay before optimal improvement could be between 6 months [33] and 2 years after the procedure [10], [12], [13], [15] and may be different for hip and knee arthroplasty. In this study, assuming a normal distribution of procedure, about one-quarter of patients may be in the period before optimal improvement. Moreover, recent hip arthroplasty may comprise cases of hip fracture. Inclusion of this sub-group in the analysis may contribute to a higher reporting of disability for the recent hip arthroplasty group. Testing 4 different categories of walking distance may be inappropriate because tests are not independent. However, we found interesting to report these 4 categories to better inform clinicians and patients. We did not develop any strategies to take into account multiple testing; some of our results may have occurred by chance. All these limitations are counterbalanced by the fact that this national survey provides both a detailed description of disabilities over time for the entire knee arthroplasty population, which consequently reflects functional status, and a comparison of activity limitations for people with recent knee arthroplasty and those with recent hip arthroplasty.

People with knee arthroplasty reported a higher risk of disability than the general population for common activities of daily living but a similar evolution. There was no relevant difference between recent knee and hip arthroplasties for mobility. This national longitudinal survey may help clinicians and other clinical practitioners such as occupational therapists, physical therapists and service planners think about specific rehabilitation programs to reduce disability according to the type of arthroplasty, hip or knee.
